# Supplementary material for: Determinants of intimate partner violence against women in Ethiopia: A multi-level analysis
Source: PLoS One. 2020 Apr 24;15(4):e0232217. doi: 10.1371/journal.pone.0232217 (PMC7182270; doi:10.1371/journal.pone.0232217)
Supplement: Attachment — Submitted filename: Response to Reviewers.docx [file pone.0232217.s001.docx]

## Responses to the reviewer comments (Manuscript ID: PONE-D-19-32625)

Dear editor and reviewers,

We would like to thank you for your careful review of the draft of our manuscript entitled “Determinants of Intimate Partner Violence against Women in Ethiopia: A Multi-Level Analysis” and providing us with advice to improve the quality of the manuscript. The response to address the comments has been prepared in the following summary table.

Sincerely,

Tenaw (on behalf of the authors)

| **Editor’s comments** | **Authors’ response** |
| --- | --- |
| Refer to the STROBE checklist and attach a copy to the next submission | We have attached the completed STROBE checklist with the revised submission. |
| Ensure the data availability statement have been carefully copy-edited | We previously stated in the submission system that we used data from the Ethiopian Demographic and Health Survey (EDHS) Program <https://dhsprogram.com/data/>. Because data was provided by a third party, we cannot share the data publicly. This has also been stated in the submission system. |
| Your ethics statement must appear in the Methods section | The following ethics statement has now been included to the methods section. “*The original survey was conducted after being ethically approved by the National Research Ethics Review Committee (NRERC) of Ethiopia (Ref. No: 3.10/114/2016). Prior to analysis, we obtained permission from the Demographic and Health Survey program and ethical approval from University of Newcastle Human Research Ethics Committee (Ref. No: H-2018-0055).*” |
| **Reviewer #1 comments** | **Authors’ response** |
| Pg. 6: The authors can present the items used to measure IPV in a table to improve the flow of the section. | IPV measures have now been presented in a Table 1. As a result, numbers of subsequent tables have changed. |
| Pg. 9, lines 151-55: The authors should explain the rationale for choosing specific cut-offs for recoding continuous community level variables into binary variables. | We classified the continuous community level variables as “low” or “high” using mean values in the data (for normally distributed characteristics) and median values (for variables that were not normally distributed). We judged that such classification would assist interpretation of the results for IPV in the context of the EDHS data, and also generate similarly sized groups, optimising statistical power. |
| Pg. 11, lines 193-194: The authors should remove the question mark next to 'random effects'. There is a question mark next to random effects in Table 5 that should be removed as well. | The question marks have been removed. |
| **Reviewer #2 comments** | **Authors’ response** |
| 1) In Table 1, instead of only listing level 1 variables, the authors should list all variables considered in the analysis and indicate at which level each variable has been used. This will make it easier for the reader to follow their description of variables used in the model and the modelling hierarchy. | This table (which is now Table 2) now lists all level 2 and level 3 variables used in the analysis. |
| 2) Authors need to provide information on how the 3897 women are distributed across the 639 communities (PSUs). | The following statement has been added in the data processing and analysis section “*On average, each cluster/community had 6 women (range, 2 to 13)*” |
| 3) Authors need to comment on the rationale behind having “Attitude to IPV” at level 1 and “Acceptance of IPV” at level 2. | We used the phrase “Attitude to IPV” as it appeared in the Ethiopian Demographic and Health Survey (EDHS) reporting. However, when we constructed the community level variable, we rephrased this as “Acceptance of IPV”. In doing so, we assumed that when individual’s attitude towards IPV is shared with in the larger community, there might be a sense of commonness/normative understanding towards IPV that could best be explained with a phrase ‘community’s acceptance of IPV’. Otherwise, both phrases represent attitudes which support IPV against women. |
| 4) Authors have indicated that individual and relationship level variables were added as level 1 because in the EDHS only 1 woman per HH was sampled. Yet when defining some level 2 variables, they are using the some of these level 1 variables to derive level 2 variables. Could they explain the rationale informing this? What is the mathematical implication of using same variables at level 1 and 2 albeit aggregate variables? | As the reviewer pointed out, some of the level 2 variables were constructed by aggregating level 1 variables. Level 1 variables reflect what is happening for individual women in their relationships, whilst Level 2 variables represent the prevalent values in the larger community, which could have an independent influence i.e. level 2 variables represent contextual effects based on aggregated individual variables. According to the ecological framework, which we have applied in this study, some individual-level factors alone may not be sufficient to explain IPV unless these factors are communal and largely shared within the community where women live. For example, in addition to personal attitude about IPV, level of acceptance towards IPV in the community or area in which women lives also matters.  Regarding the mathematical implication of using same variables at level 1 and 2: Bafumi and Gelman have previously shown that the same variables can be included at both level 1 and level 2 of a multilevel model by including an aggregate measure of the level 1 variable at level 2. In this publication they showed that this approach can remove omitted variable bias when there is correlation between different level 1 and level 2 variables. Via simulations they show that by including the level 1 variable also at level 2 in aggregated form, the error term becomes uncorrelated with the level 1 predictor and parameters can be estimated with correct standard errors. Thus the method alleviates, rather than introduces, methodological issues [1]. |
| 5) If level 2 and level 3 variables were aggregated at community and provincial level respectively, the summary statistics presented in the Tables 2 and 3 for Level 2 and 3 variables should reflect the sample size at that level (n = 639 for level 2 and 11 for level 3) | The summary statistics have now been reported at level 2 and level 3 based on the number of sampling units (communities and regions, respectively) at each level. |
| 6) Authors should comment on results in Table 3. | To comment on these results, we added the statement “*Table 4 shows IPV experience by different variables*”. Since detailed explanations were already provided for the univariate association between variables and the outcome (IPV) from Table 5, we did not add further explanations from Table 4. |
| 7) Authors should comment on how they decided on cut-offs for community level variables. | We previously mentioned that the community level variables were constructed by aggregating individual- or relationship- level characteristics. For example, early marriage in the community was defined as proportion of women in that community who married before 18 years of age. The variables were then grouped into lower and higher categories using mean (for normally distributed characteristics) and median (for variables that were not normally distributed) values. In the case of early marriage, the distribution was right skewed and the median of the proportion of women in the community who married before 18 years of age was 60%. Then, early marriage in the community was categorized as high if the proportion of women married before 18 years of age was 60.0-100% and low if the proportion was 0-59.9%. This is shown at pg. 9, lines 147-151 of the original submission. |
| 8) Association between age of women and lifetime experience of IPV is always difficult to entangle. I would suggest that the authors use the participant’s actual age (continuous var) as a covariate in the models rather than dwell on discussing this as possible determinant of IPV. The authors could look at whether they will get different outcome if they include age (continuous var). | We repeated the analysis with the ‘continuous age variable’ and we found $\beta=1.026111, p <0.001$ in the final model. However, we just wanted to discuss some social roles, expectations and/or responsibilities in Ethiopia associated with old and/or young age that could be a risk or protective of IPV against women. The five-year age interval classification of women’s reproductive years (15-49) is widely used in previous IPV papers and we followed the same approach. For example, a secondary analysis of Demographic and Health Survey (DHS) data from nine low and middle income countries [2] and a report from the analysis of DHS data from 10 countries [3] have used the five-year age classification. Small scale studies in Ethiopia on the determinants of IPV have also followed the analysis approach of using the categorical age variable [4-6]. |
| 9) In Line 319-320, is there evidence in the data that most of the uneducated women in the sample were from rural communities? | Yes, in rural areas, 69.3% of women were uneducated, while only 21.4% of women living in urban area were uneducated. We have amended the sentence and now the sentence reads, “*Finally, Ethiopian women from rural areas, in which the majority of women are uneducated, often do not know their exact age and this could contribute to discrepancies as a result of measurement error*”. |
| 10) To support their assertions in line 332-334, did the authors look at whether there was an association between acceptance of IPV and witnessing of inter-parental violence? | We have checked if there is a significant association between the two variables using a chi-square test and the following statement is added. “*Further analysis of this data using a chi-square*$test$ *also revealed that there was significant association between witnessing of inter-parental violence and IPV accepting attitude (p = 0.036)*” |
| 11) In line 346-348, it is not possible to attribute causality with a cross-sectional study (as they have acknowledged in their limitations). Do not think the reason for non-significant relationship is due to assumed direction of effect. The association between factors is not dependent on what you define as an outcome. | We agree with the reviewer and have changed this statement to: “*In this study, women’s own substance abuse was not found to be significantly associated with IPV. This needs further investigation as the relationship between women’s substance abuse and her experience of IPV is often complex. There are also views that women use substances in response to experiences of IPV, rather than their substance use being a reason of IPV.*” |
| 12) Considering almost 60 % of the women and 50% of their partners have no education, it would be interesting to understand the distribution of the differential education (especially which women have high education than their partners). | This is an interesting comment. We would appreciate the reviewer suggesting which characteristics they would like to see the distribution of education by. For example, we looked at the distribution of differential education across rurality, age, employment, and wealth index. The data revealed that of 379 women who have higher educational attainment than their partners, most of them were from rural area (n = 301, 79.4%), in the age group 20-24 years (n = 109, 28.7%), employed (n = 207, 54.7%), and in the richest household (n = 81, 21.3%). One of the interesting finding is that despite most (69.3%) of rural women were uneducated, those who were educated were also married to uneducated partners i.e. most (79.4%) of women who have higher educational attainment than their partners were from rural area. |
| 13) The authors should also highlight the limitations of using secondary data vis a vis their ecological model. Were there other factors they could have included that were not available in the dataset? | The following limitation has been added in the limitation section. “*Lastly, some factors of the ecological model such as factors related to social support to victims, neighbourhood environment, laws, and national policies were not assessed in this study due to these variables not being in the dataset.*” |
| 14) Overall the discussion section is too long. They should make it more succinct. | Yes, we agree that due to the many variables we assessed, the discussion is a bit long. We have now edited the discussion to make it more concise, emphasising the key findings. |

**References**

1.	Bafumi, J. and A. Gelman, *Fitting multilevel models when predictors and group effects correlate.* Available at SSRN 1010095.

2.	Sunita, K. and J. Kiersten, *Profiling Domestic Violence: A Multi-Country Study*. 2004: Calverton, Maryland,ORC Macro.

3.	Hindin, M.J., S. Kishor, and D.L. Ansara, *Intimate partner violence among couples in 10 DHS countries: predictors and health outcomes.* 2008.

4.	Abeya, S.G., M.F. Afework, and A.W. Yalew, *Intimate partner violence against women in western Ethiopia: prevalence, patterns, and associated factors.* BMC Public Health, 2011. **11**: p. 913.

5.	Semahegn, A., T. Belachew, and M. Abdulahi, *Domestic violence and its predictors among married women in reproductive age in Fagitalekoma Woreda, Awi zone, Amhara regional state, North Western Ethiopia.* Reproductive Health, 2013. **10**.

6.	Dibaba, Y., *Prevalence and correlates of intimate partner physical violence against women in Kofale district Ethiopia.* Tropical Doctor, 2008. **38**(1): p. 52-54.
